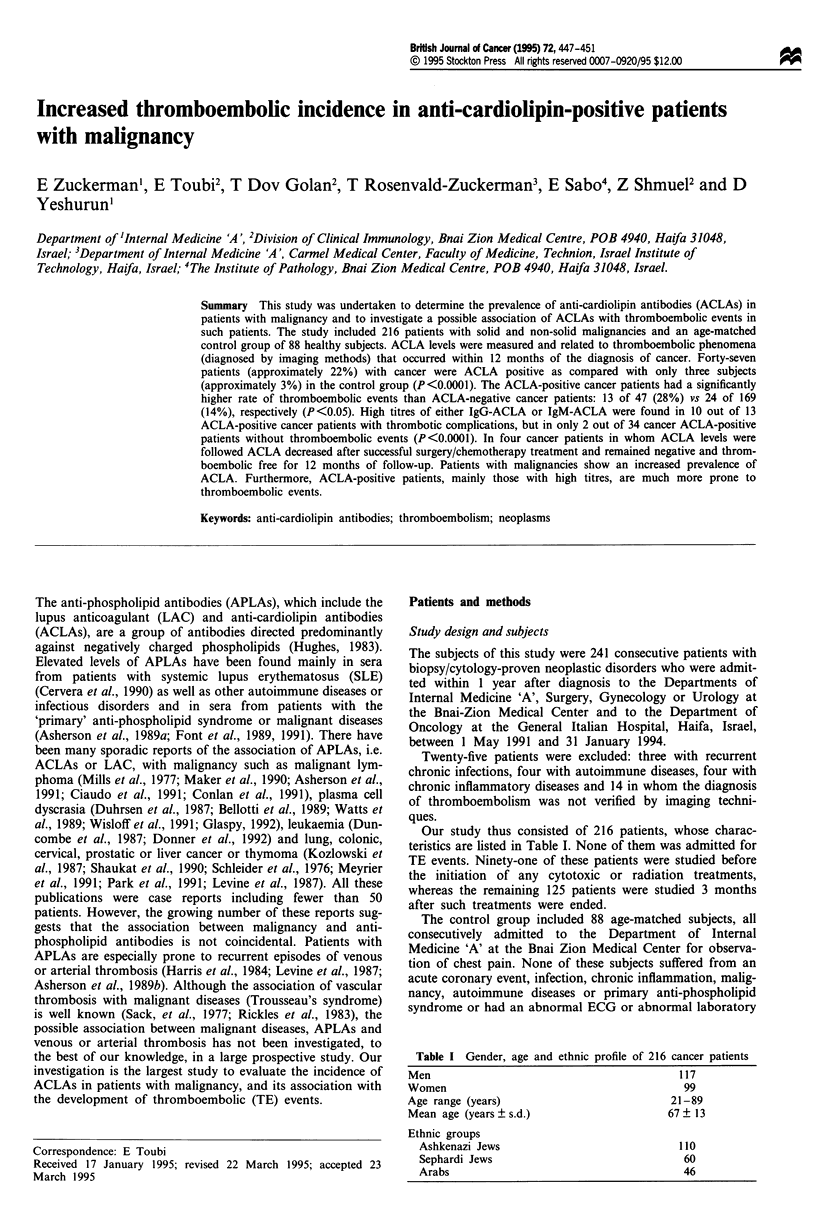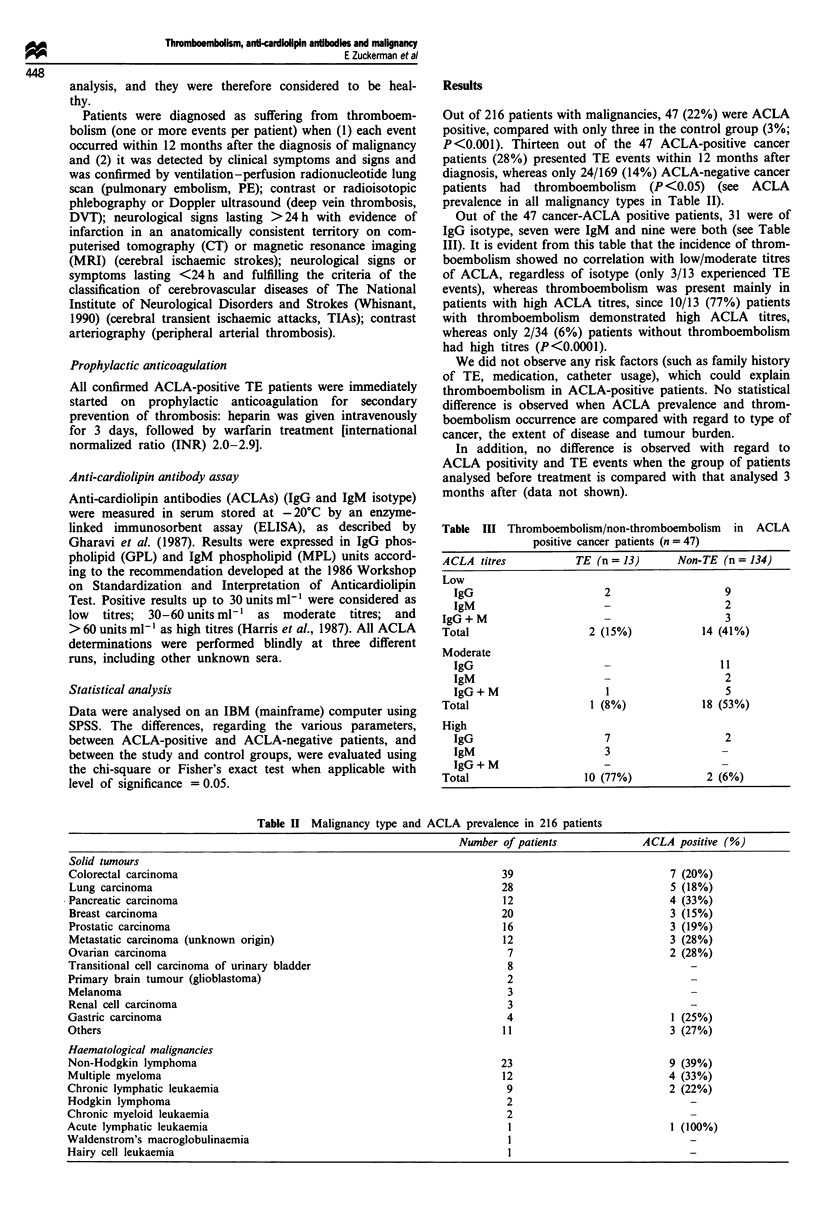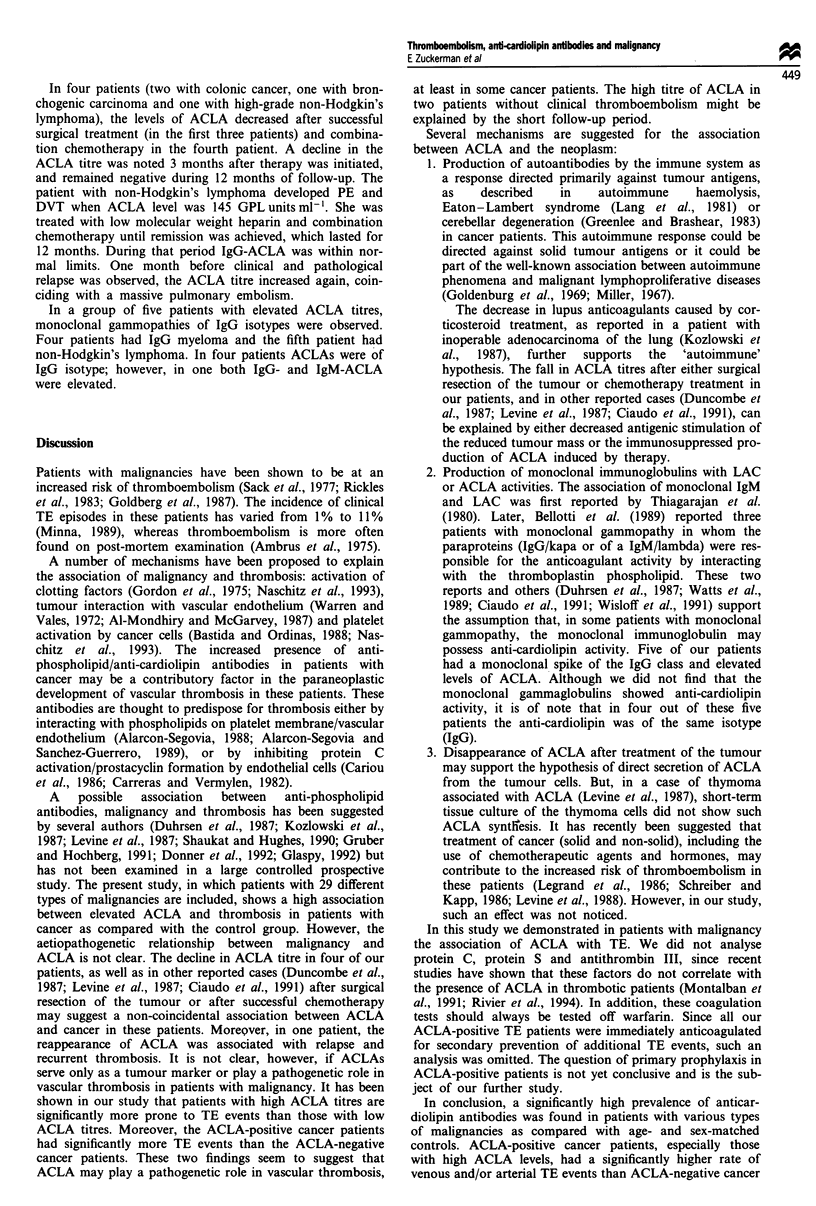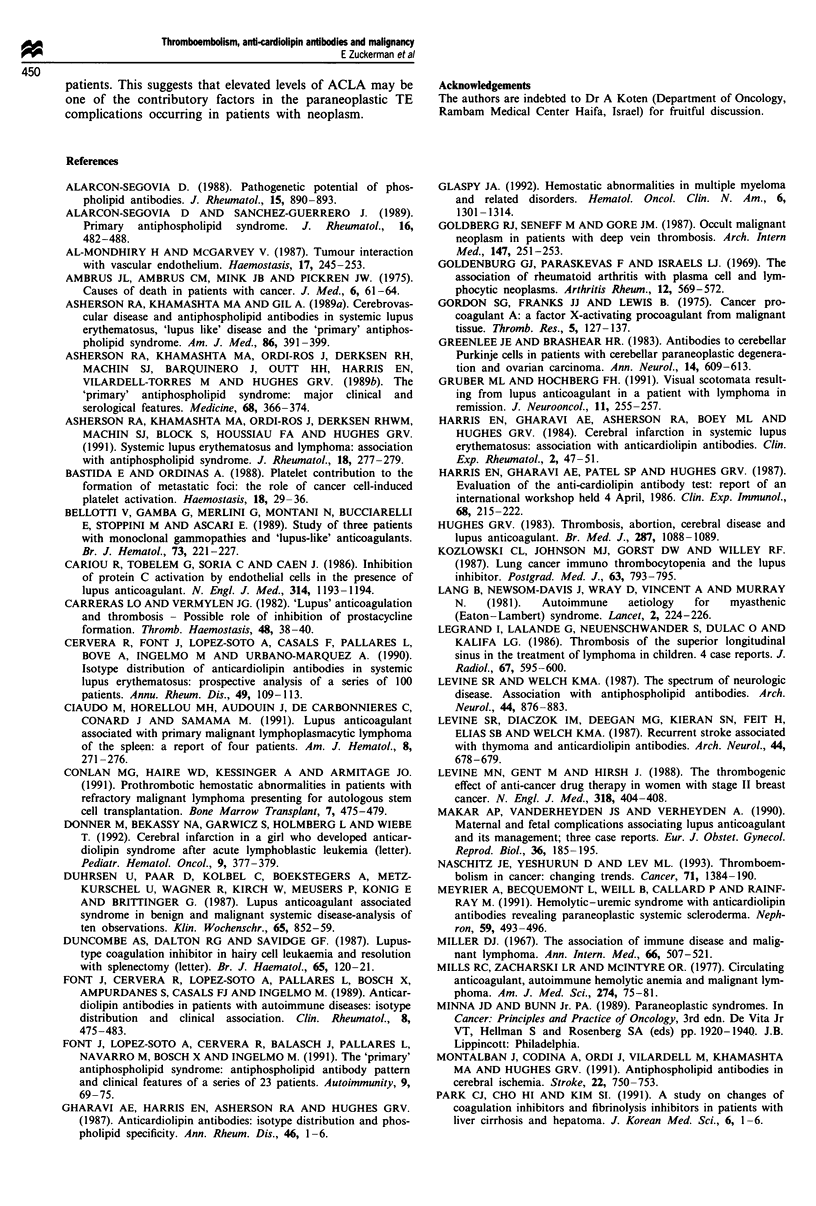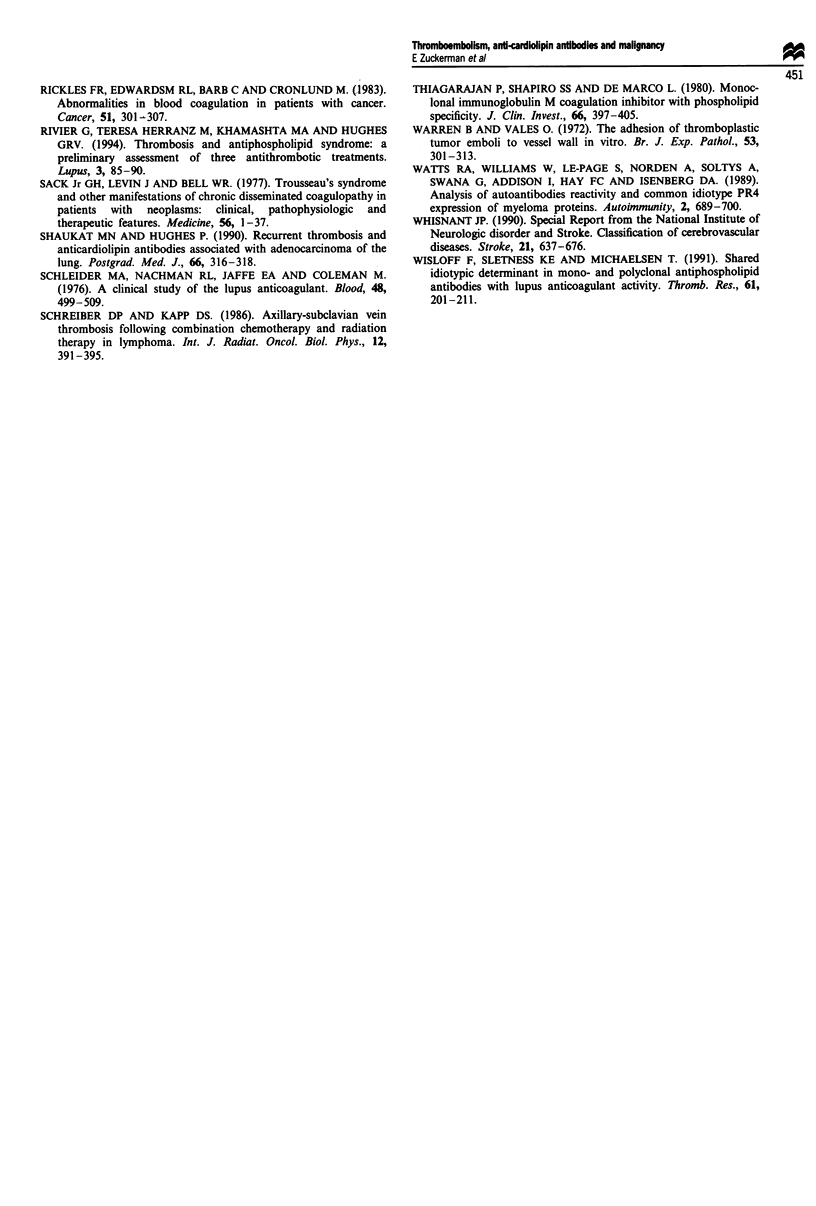# Increased thromboembolic incidence in anti-cardiolipin-positive patients with malignancy.

**DOI:** 10.1038/bjc.1995.353

**Published:** 1995-08

**Authors:** E. Zuckerman, E. Toubi, T. D. Golan, T. Rosenvald-Zuckerman, E. Sabo, Z. Shmuel, D. Yeshurun

**Affiliations:** Department of Internal Medicine A, Bnai Zion Medical Centre, Haifa, Israel.

## Abstract

This study was undertaken to determine the prevalence of anti-cardiolipin antibodies (ACLAs) in patients with malignancy and to investigate a possible association of ACLAs with thromboembolic events in such patients. The study included 216 patients with solid and non-solid malignancies and an age-matched control group of 88 healthy subjects. ACLA levels were measured and related to thromboembolic phenomena (diagnosed by imaging methods) that occurred within 12 months of the diagnosis of cancer. Forty-seven patients (approximately 22%) with cancer were ACLA positive as compared with only three subjects (approximately 3%) in the control group (P < 0.0001). The ACLA-positive cancer patients had a significantly higher rate of thromboembolic events than ACLA-negative cancer patients: 13 of 47 (28%) vs 24 of 169 (14%), respectively (P < 0.05). High titres of either IgG-ACLA or IgM-ACLA were found in 10 out of 13 ACLA-positive cancer patients with thrombotic complications, but in only 2 out of 34 cancer ACLA-positive patients without thromboembolic events (P < 0.0001). In four cancer patients in whom ACLA levels were followed ACLA decreased after successful surgery/chemotherapy treatment and remained negative and thromboembolic free for 12 months of follow-up. Patients with malignancies show an increased prevalence of ACLA. Furthermore, ACLA-positive patients, mainly those with high titres, are much more prone to thromboembolic events.


British Journal of Cancer (1995) 72, 447-451
© 1995 Stockton Press All rights reserved 0007-0920/95 $12.00

### Increased thromboembolic incidence in anti-cardiolipin-positive patients with malignancy

E Zuckerman[1], E Toubi[2], T Dov Golan[2], T Rosenvald-Zuckerman[3], E Sabo[4], Z Shmuel[2] and D Yeshurun[1]

*Department of [1]Internal Medicine 'A', [2]Division of Clinical Immunology, Bnai Zion Medical Centre, POB 4940, Haifa 31048, Israel; [3]Department of Internal Medicine 'A', Carmel Medical Center, Faculty of Medicine, Technion, Israel Institute of Technology, Haifa, Israel; [4]The Institute of Pathology, Bnai Zion Medical Centre, POB 4940, Haifa 31048, Israel.*

**Summary** This study was undertaken to determine the prevalence of anti-cardiolipin antibodies (ACLAs) in patients with malignancy and to investigate a possible association of ACLAs with thromboembolic events in such patients. The study included 216 patients with solid and non-solid malignancies and an age-matched control group of 88 healthy subjects. ACLA levels were measured and related to thromboembolic phenomena (diagnosed by imaging methods) that occurred within 12 months of the diagnosis of cancer. Forty-seven patients (approximately 22%) with cancer were ACLA positive as compared with only three subjects (approximately 3%) in the control group ($P<0.0001$). The ACLA-positive cancer patients had a significantly higher rate of thromboembolic events than ACLA-negative cancer patients: 13 of 47 (28%) *vs* 24 of 169 (14%), respectively ($P<0.05$). High titres of either IgG-ACLA or IgM-ACLA were found in 10 out of 13 ACLA-positive cancer patients with thrombotic complications, but in only 2 out of 34 cancer ACLA-positive patients without thromboembolic events ($P<0.0001$). In four cancer patients in whom ACLA levels were followed ACLA decreased after successful surgery/chemotherapy treatment and remained negative and thromboembolic free for 12 months of follow-up. Patients with malignancies show an increased prevalence of ACLA. Furthermore, ACLA-positive patients, mainly those with high titres, are much more prone to thromboembolic events.

**Keywords:** anti-cardiolipin antibodies; thromboembolism; neoplasms

The anti-phospholipid antibodies (APLAs), which include the lupus anticoagulant (LAC) and anti-cardiolipin antibodies (ACLAs), are a group of antibodies directed predominantly against negatively charged phospholipids (Hughes, 1983). Elevated levels of APLAs have been found mainly in sera from patients with systemic lupus erythematosus (SLE) (Cervera *et al.*, 1990) as well as other autoimmune diseases or infectious disorders and in sera from patients with the 'primary' anti-phospholipid syndrome or malignant diseases (Asherson *et al.*, 1989*a*; Font *et al.*, 1989, 1991). There have been many sporadic reports of the association of APLAs, i.e. ACLAs or LAC, with malignancy such as malignant lymphoma (Mills *et al.*, 1977; Maker *et al.*, 1990; Asherson *et al.*, 1991; Ciaudo *et al.*, 1991; Conlan *et al.*, 1991), plasma cell dyscrasia (Duhrsen *et al.*, 1987; Bellotti *et al.*, 1989; Watts *et al.*, 1989; Wisloff *et al.*, 1991; Glaspy, 1992), leukaemia (Duncombe *et al.*, 1987; Donner *et al.*, 1992) and lung, colonic, cervical, prostatic or liver cancer or thymoma (Kozlowski *et al.*, 1987; Shaukat *et al.*, 1990; Schleider *et al.*, 1976; Meyrier *et al.*, 1991; Park *et al.*, 1991; Levine *et al.*, 1987). All these publications were case reports including fewer than 50 patients. However, the growing number of these reports suggests that the association between malignancy and anti-phospholipid antibodies is not coincidental. Patients with APLAs are especially prone to recurrent episodes of venous or arterial thrombosis (Harris *et al.*, 1984; Levine *et al.*, 1987; Asherson *et al.*, 1989*b*). Although the association of vascular thrombosis with malignant diseases (Trousseau's syndrome) is well known (Sack, *et al.*, 1977; Rickles *et al.*, 1983), the possible association between malignant diseases, APLAs and venous or arterial thrombosis has not been investigated, to the best of our knowledge, in a large prospective study. Our investigation is the largest study to evaluate the incidence of ACLAs in patients with malignancy, and its association with the development of thromboembolic (TE) events.

Correspondence: E Toubi
Received 17 January 1995; revised 22 March 1995; accepted 23 March 1995

### Patients and methods

### *Study design and subjects*

The subjects of this study were 241 consecutive patients with biopsy/cytology-proven neoplastic disorders who were admitted within 1 year after diagnosis to the Departments of Internal Medicine 'A', Surgery, Gynecology or Urology at the Bnai-Zion Medical Center and to the Department of Oncology at the General Italian Hospital, Haifa, Israel, between 1 May 1991 and 31 January 1994.

Twenty-five patients were excluded: three with recurrent chronic infections, four with autoimmune diseases, four with chronic inflammatory diseases and 14 in whom the diagnosis of thromboembolism was not verified by imaging techniques.

Our study thus consisted of 216 patients, whose characteristics are listed in Table I. None of them was admitted for TE events. Ninety-one of these patients were studied before the initiation of any cytotoxic or radiation treatments, whereas the remaining 125 patients were studied 3 months after such treatments were ended.

The control group included 88 age-matched subjects, all consecutively admitted to the Department of Internal Medicine 'A' at the Bnai Zion Medical Center for observation of chest pain. None of these subjects suffered from an acute coronary event, infection, chronic inflammation, malignancy, autoimmune diseases or primary anti-phospholipid syndrome or had an abnormal ECG or abnormal laboratory

**Table I** Gender, age and ethnic profile of 216 cancer patients

| | |
|---|---|
| Men | 117 |
| Women | 99 |
| Age range (years) | 21-89 |
| Mean age (years ± s.d.) | 67 ± 13 |
| Ethnic groups | |
| Ashkenazi Jews | 110 |
| Sephardi Jews | 60 |
| Arabs | 46 |

analysis, and they were therefore considered to be healthy.

Patients were diagnosed as suffering from thromboembolism (one or more events per patient) when (1) each event occurred within 12 months after the diagnosis of malignancy and (2) it was detected by clinical symptoms and signs and was confirmed by ventilation–perfusion radionucleotide lung scan (pulmonary embolism, PE); contrast or radioisotopic phlebography or Doppler ultrasound (deep vein thrombosis, DVT); neurological signs lasting >24 h with evidence of infarction in an anatomically consistent territory on computerised tomography (CT) or magnetic resonance imaging (MRI) (cerebral ischaemic strokes); neurological signs or symptoms lasting <24 h and fulfilling the criteria of the classification of cerebrovascular diseases of The National Institute of Neurological Disorders and Strokes (Whisnant, 1990) (cerebral transient ischaemic attacks, TIAs); contrast arteriography (peripheral arterial thrombosis).

### *Prophylactic anticoagulation*

All confirmed ACLA-positive TE patients were immediately started on prophylactic anticoagulation for secondary prevention of thrombosis: heparin was given intravenously for 3 days, followed by warfarin treatment [international normalized ratio (INR) 2.0–2.9].

### *Anti-cardiolipin antibody assay*

Anti-cardiolipin antibodies (ACLAs) (IgG and IgM isotype) were measured in serum stored at −20°C by an enzyme-linked immunosorbent assay (ELISA), as described by Gharavi *et al.* (1987). Results were expressed in IgG phospholipid (GPL) and IgM phospholipid (MPL) units according to the recommendation developed at the 1986 Workshop on Standardization and Interpretation of Anticardiolipin Test. Positive results up to 30 units $ml^{-1}$ were considered as low titres; 30–60 units $ml^{-1}$ as moderate titres; and >60 units $ml^{-1}$ as high titres (Harris *et al.*, 1987). All ACLA determinations were performed blindly at three different runs, including other unknown sera.

### *Statistical analysis*

Data were analysed on an IBM (mainframe) computer using SPSS. The differences, regarding the various parameters, between ACLA-positive and ACLA-negative patients, and between the study and control groups, were evaluated using the chi-square or Fisher's exact test when applicable with level of significance =0.05.

### Results

Out of 216 patients with malignancies, 47 (22%) were ACLA positive, compared with only three in the control group (3%; $P<0.001$). Thirteen out of the 47 ACLA-positive cancer patients (28%) presented TE events within 12 months after diagnosis, whereas only 24/169 (14%) ACLA-negative cancer patients had thromboembolism ($P<0.05$) (see ACLA prevalence in all malignancy types in Table II).

Out of the 47 cancer-ACLA positive patients, 31 were of IgG isotype, seven were IgM and nine were both (see Table III). It is evident from this table that the incidence of thromboembolism showed no correlation with low/moderate titres of ACLA, regardless of isotype (only 3/13 experienced TE events), whereas thromboembolism was present mainly in patients with high ACLA titres, since 10/13 (77%) patients with thromboembolism demonstrated high ACLA titres, whereas only 2/34 (6%) patients without thromboembolism had high titres ($P<0.0001$).

We did not observe any risk factors (such as family history of TE, medication, catheter usage), which could explain thromboembolism in ACLA-positive patients. No statistical difference is observed when ACLA prevalence and thromboembolism occurrence are compared with regard to type of cancer, the extent of disease and tumour burden.

In addition, no difference is observed with regard to ACLA positivity and TE events when the group of patients analysed before treatment is compared with that analysed 3 months after (data not shown).

**Table III** Thromboembolism/non-thromboembolism in ACLA positive cancer patients ($n=47$)

| *ACLA titres* | *TE* (n = *13*) | *Non-TE* (n = *134*) |
|---|---|---|
| Low | | |
| IgG | 2 | 9 |
| IgM | – | 2 |
| IgG + M | – | 3 |
| Total | 2 (15%) | 14 (41%) |
| Moderate | | |
| IgG | – | 11 |
| IgM | – | 2 |
| IgG + M | 1 | 5 |
| Total | 1 (8%) | 18 (53%) |
| High | | |
| IgG | 7 | 2 |
| IgM | 3 | – |
| IgG + M | – | – |
| Total | 10 (77%) | 2 (6%) |

**Table II** Malignancy type and ACLA prevalence in 216 patients

| | *Number of patients* | *ACLA positive (%)* |
|---|---|---|
| *Solid tumours* | | |
| Colorectal carcinoma | 39 | 7 (20%) |
| Lung carcinoma | 28 | 5 (18%) |
| Pancreatic carcinoma | 12 | 4 (33%) |
| Breast carcinoma | 20 | 3 (15%) |
| Prostatic carcinoma | 16 | 3 (19%) |
| Metastatic carcinoma (unknown origin) | 12 | 3 (28%) |
| Ovarian carcinoma | 7 | 2 (28%) |
| Transitional cell carcinoma of urinary bladder | 8 | – |
| Primary brain tumour (glioblastoma) | 2 | – |
| Melanoma | 3 | – |
| Renal cell carcinoma | 3 | – |
| Gastric carcinoma | 4 | 1 (25%) |
| Others | 11 | 3 (27%) |
| *Haematological malignancies* | | |
| Non-Hodgkin lymphoma | 23 | 9 (39%) |
| Multiple myeloma | 12 | 4 (33%) |
| Chronic lymphatic leukaemia | 9 | 2 (22%) |
| Hodgkin lymphoma | 2 | – |
| Chronic myeloid leukaemia | 2 | – |
| Acute lymphatic leukaemia | 1 | 1 (100%) |
| Waldenstrom's macroglobulinaemia | 1 | – |
| Hairy cell leukaemia | 1 | – |

In four patients (two with colonic cancer, one with bronchogenic carcinoma and one with high-grade non-Hodgkin's lymphoma), the levels of ACLA decreased after successful surgical treatment (in the first three patients) and combination chemotherapy in the fourth patient. A decline in the ACLA titre was noted 3 months after therapy was initiated, and remained negative during 12 months of follow-up. The patient with non-Hodgkin's lymphoma developed PE and DVT when ACLA level was 145 GPL units $ml^{-1}$. She was treated with low molecular weight heparin and combination chemotherapy until remission was achieved, which lasted for 12 months. During that period IgG-ACLA was within normal limits. One month before clinical and pathological relapse was observed, the ACLA titre increased again, coinciding with a massive pulmonary embolism.

In a group of five patients with elevated ACLA titres, monoclonal gammopathies of IgG isotypes were observed. Four patients had IgG myeloma and the fifth patient had non-Hodgkin's lymphoma. In four patients ACLAs were of IgG isotype; however, in one both IgG- and IgM-ACLA were elevated.

### Discussion

Patients with malignancies have been shown to be at an increased risk of thromboembolism (Sack *et al.*, 1977; Rickles *et al.*, 1983; Goldberg *et al.*, 1987). The incidence of clinical TE episodes in these patients has varied from 1% to 11% (Minna, 1989), whereas thromboembolism is more often found on post-mortem examination (Ambrus *et al.*, 1975).

A number of mechanisms have been proposed to explain the association of malignancy and thrombosis: activation of clotting factors (Gordon *et al.*, 1975; Naschitz *et al.*, 1993), tumour interaction with vascular endothelium (Warren and Vales, 1972; Al-Mondhiry and McGarvey, 1987) and platelet activation by cancer cells (Bastida and Ordinas, 1988; Naschitz *et al.*, 1993). The increased presence of anti-phospholipid/anti-cardiolipin antibodies in patients with cancer may be a contributory factor in the paraneoplastic development of vascular thrombosis in these patients. These antibodies are thought to predispose for thrombosis either by interacting with phospholipids on platelet membrane/vascular endothelium (Alarcon-Segovia, 1988; Alarcon-Segovia and Sanchez-Guerrero, 1989), or by inhibiting protein C activation/prostacyclin formation by endothelial cells (Cariou *et al.*, 1986; Carreras and Vermylen, 1982).

A possible association between anti-phospholipid antibodies, malignancy and thrombosis has been suggested by several authors (Duhrsen *et al.*, 1987; Kozlowski *et al.*, 1987; Levine *et al.*, 1987; Shaukat and Hughes, 1990; Gruber and Hochberg, 1991; Donner *et al.*, 1992; Glaspy, 1992) but has not been examined in a large controlled prospective study. The present study, in which patients with 29 different types of malignancies are included, shows a high association between elevated ACLA and thrombosis in patients with cancer as compared with the control group. However, the aetiopathogenetic relationship between malignancy and ACLA is not clear. The decline in ACLA titre in four of our patients, as well as in other reported cases (Duncombe *et al.*, 1987; Levine *et al.*, 1987; Ciaudo *et al.*, 1991) after surgical resection of the tumour or after successful chemotherapy may suggest a non-coincidental association between ACLA and cancer in these patients. Moreover, in one patient, the reappearance of ACLA was associated with relapse and recurrent thrombosis. It is not clear, however, if ACLAs serve only as a tumour marker or play a pathogenetic role in vascular thrombosis in patients with malignancy. It has been shown in our study that patients with high ACLA titres are significantly more prone to TE events than those with low ACLA titres. Moreover, the ACLA-positive cancer patients had significantly more TE events than the ACLA-negative cancer patients. These two findings seem to suggest that ACLA may play a pathogenetic role in vascular thrombosis, at least in some cancer patients. The high titre of ACLA in two patients without clinical thromboembolism might be explained by the short follow-up period.

Several mechanisms are suggested for the association between ACLA and the neoplasm:

1. Production of autoantibodies by the immune system as a response directed primarily against tumour antigens, as described in autoimmune haemolysis, Eaton–Lambert syndrome (Lang *et al.*, 1981) or cerebellar degeneration (Greenlee and Brashear, 1983) in cancer patients. This autoimmune response could be directed against solid tumour antigens or it could be part of the well-known association between autoimmune phenomena and malignant lymphoproliferative diseases (Goldenburg *et al.*, 1969; Miller, 1967).

   The decrease in lupus anticoagulants caused by corticosteroid treatment, as reported in a patient with inoperable adenocarcinoma of the lung (Kozlowski *et al.*, 1987), further supports the 'autoimmune' hypothesis. The fall in ACLA titres after either surgical resection of the tumour or chemotherapy treatment in our patients, and in other reported cases (Duncombe *et al.*, 1987; Levine *et al.*, 1987; Ciaudo *et al.*, 1991), can be explained by either decreased antigenic stimulation of the reduced tumour mass or the immunosuppressed production of ACLA induced by therapy.
2. Production of monoclonal immunoglobulins with LAC or ACLA activities. The association of monoclonal IgM and LAC was first reported by Thiagarajan *et al.* (1980). Later, Bellotti *et al.* (1989) reported three patients with monoclonal gammopathy in whom the paraproteins (IgG/kapa or of a IgM/lambda) were responsible for the anticoagulant activity by interacting with the thromboplastin phospholipid. These two reports and others (Duhrsen *et al.*, 1987; Watts *et al.*, 1989; Ciaudo *et al.*, 1991; Wisloff *et al.*, 1991) support the assumption that, in some patients with monoclonal gammopathy, the monoclonal immunoglobulin may possess anti-cardiolipin activity. Five of our patients had a monoclonal spike of the IgG class and elevated levels of ACLA. Although we did not find that the monoclonal gammaglobulins showed anti-cardiolipin activity, it is of note that in four out of these five patients the anti-cardiolipin was of the same isotype (IgG).
3. Disappearance of ACLA after treatment of the tumour may support the hypothesis of direct secretion of ACLA from the tumour cells. But, in a case of thymoma associated with ACLA (Levine *et al.*, 1987), short-term tissue culture of the thymoma cells did not show such ACLA synthesis. It has recently been suggested that treatment of cancer (solid and non-solid), including the use of chemotherapeutic agents and hormones, may contribute to the increased risk of thromboembolism in these patients (Legrand *et al.*, 1986; Schreiber and Kapp, 1986; Levine *et al.*, 1988). However, in our study, such an effect was not noticed.

In this study we demonstrated in patients with malignancy the association of ACLA with TE. We did not analyse protein C, protein S and antithrombin III, since recent studies have shown that these factors do not correlate with the presence of ACLA in thrombotic patients (Montalban *et al.*, 1991; Rivier *et al.*, 1994). In addition, these coagulation tests should always be tested off warfarin. Since all our ACLA-positive TE patients were immediately anticoagulated for secondary prevention of additional TE events, such an analysis was omitted. The question of primary prophylaxis in ACLA-positive patients is not yet conclusive and is the subject of our further study.

In conclusion, a significantly high prevalence of anticardiolipin antibodies was found in patients with various types of malignancies as compared with age- and sex-matched controls. ACLA-positive cancer patients, especially those with high ACLA levels, had a significantly higher rate of venous and/or arterial TE events than ACLA-negative cancer patients. This suggests that elevated levels of ACLA may be one of the contributory factors in the paraneoplastic TE complications occurring in patients with neoplasm.

**Acknowledgements**
The authors are indebted to Dr A Koten (Department of Oncology, Rambam Medical Center Haifa, Israel) for fruitful discussion.

### References

ALARCON-SEGOVIA D. (1988). Pathogenetic potential of phospholipid antibodies. *J. Rheumatol.*, **15**, 890–893.

ALARCON-SEGOVIA D AND SANCHEZ-GUERRERO J. (1989). Primary antiphospholipid syndrome. *J. Rheumatol.*, **16**, 482–488.

AL-MONDHIRY H AND MCGARVEY V. (1987). Tumour interaction with vascular endothelium. *Haemostasis*, **17**, 245–253.

AMBRUS JL, AMBRUS CM, MINK JB AND PICKREN JW. (1975). Causes of death in patients with cancer. *J. Med.*, **6**, 61–64.

ASHERSON RA, KHAMASHTA MA AND GIL A. (1989*a*). Cerebrovascular disease and antiphospholipid antibodies in systemic lupus erythematosus, 'lupus like' disease and the 'primary' antiphospholipid syndrome. *Am. J. Med.*, **86**, 391–399.

ASHERSON RA, KHAMASHTA MA, ORDI-ROS J, DERKSEN RH, MACHIN SJ, BARQUINERO J, OUTT HH, HARRIS EN, VILARDELL-TORRES M AND HUGHES GRV. (1989*b*). The 'primary' antiphospholipid syndrome: major clinical and serological features. *Medicine*, **68**, 366–374.

ASHERSON RA, KHAMASHTA MA, ORDI-ROS J, DERKSEN RHWM, MACHIN SJ, BLOCK S, HOUSSIAU FA AND HUGHES GRV. (1991). Systemic lupus erythematosus and lymphoma: association with antiphospholipid syndrome. *J. Rheumatol.*, **18**, 277–279.

BASTIDA E AND ORDINAS A. (1988). Platelet contribution to the formation of metastatic foci: the role of cancer cell-induced platelet activation. *Haemostasis*, **18**, 29–36.

BELLOTTI V, GAMBA G, MERLINI G, MONTANI N, BUCCIARELLI E, STOPPINI M AND ASCARI E. (1989). Study of three patients with monoclonal gammopathies and 'lupus-like' anticoagulants. *Br. J. Hematol.*, **73**, 221–227.

CARIOU R, TOBELEM G, SORIA C AND CAEN J. (1986). Inhibition of protein C activation by endothelial cells in the presence of lupus anticoagulant. *N. Engl. J. Med.*, **314**, 1193–1194.

CARRERAS LO AND VERMYLEN JG. (1982). 'Lupus' anticoagulation and thrombosis – Possible role of inhibition of prostacycline formation. *Thromb. Haemostasis*, **48**, 38–40.

CERVERA R, FONT J, LOPEZ-SOTO A, CASALS F, PALLARES L, BOVE A, INGELMO M AND URBANO-MARQUEZ A. (1990). Isotype distribution of anticardiolipin antibodies in systemic lupus erythematosus: prospective analysis of a series of 100 patients. *Annu. Rheum. Dis.*, **49**, 109–113.

CIAUDO M, HORELLOU MH, AUDOUIN J, DE CARBONNIERES C, CONARD J AND SAMAMA M. (1991). Lupus anticoagulant associated with primary malignant lymphoplasmacytic lymphoma of the spleen: a report of four patients. *Am. J. Hematol.*, **8**, 271–276.

CONLAN MG, HAIRE WD, KESSINGER A AND ARMITAGE JO. (1991). Prothrombotic hemostatic abnormalities in patients with refractory malignant lymphoma presenting for autologous stem cell transplantation. *Bone Marrow Transplant*, **7**, 475–479.

DONNER M, BEKASSY NA, GARWICZ S, HOLMBERG L AND WIEBE T. (1992). Cerebral infarction in a girl who developed anticardiolipin syndrome after acute lymphoblastic leukemia (letter). *Pediatr. Hematol. Oncol.*, **9**, 377–379.

DUHRSEN U, PAAR D, KOLBEL C, BOEKSTEGERS A, METZ-KURSCHEL U, WAGNER R, KIRCH W, MEUSERS P, KONIG E AND BRITTINGER G. (1987). Lupus anticoagulant associated syndrome in benign and malignant systemic disease-analysis of ten observations. *Klin. Wochenschr.*, **65**, 852–59.

DUNCOMBE AS, DALTON RG AND SAVIDGE GF. (1987). Lupus-type coagulation inhibitor in hairy cell leukaemia and resolution with splenectomy (letter). *Br. J. Haematol.*, **65**, 120–21.

FONT J, CERVERA R, LOPEZ-SOTO A, PALLARES L, BOSCH X, AMPURDANES S, CASALS FJ AND INGELMO M. (1989). Anticardiolipin antibodies in patients with autoimmune diseases: isotype distribution and clinical association. *Clin. Rheumatol.*, **8**, 475–483.

FONT J, LOPEZ-SOTO A, CERVERA R, BALASCH J, PALLARES L, NAVARRO M, BOSCH X AND INGELMO M. (1991). The 'primary' antiphospholipid syndrome: antiphospholipid antibody pattern and clinical features of a series of 23 patients. *Autoimmunity*, **9**, 69–75.

GHARAVI AE, HARRIS EN, ASHERSON RA AND HUGHES GRV. (1987). Anticardiolipin antibodies: isotype distribution and phospholipid specificity. *Ann. Rheum. Dis.*, **46**, 1–6.

GLASPY JA. (1992). Hemostatic abnormalities in multiple myeloma and related disorders. *Hematol. Oncol. Clin. N. Am.*, **6**, 1301–1314.

GOLDBERG RJ, SENEFF M AND GORE JM. (1987). Occult malignant neoplasm in patients with deep vein thrombosis. *Arch. Intern Med.*, **147**, 251–253.

GOLDENBURG GJ, PARASKEVAS F AND ISRAELS LJ. (1969). The association of rheumatoid arthritis with plasma cell and lymphocytic neoplasms. *Arthritis Rheum.*, **12**, 569–572.

GORDON SG, FRANKS JJ AND LEWIS B. (1975). Cancer procoagulant A: a factor X-activating procoagulant from malignant tissue. *Thromb. Res.*, **5**, 127–137.

GREENLEE JE AND BRASHEAR HR. (1983). Antibodies to cerebellar Purkinje cells in patients with cerebellar paraneoplastic degeneration and ovarian carcinoma. *Ann. Neurol.*, **14**, 609–613.

GRUBER ML AND HOCHBERG FH. (1991). Visual scotomata resulting from lupus anticoagulant in a patient with lymphoma in remission. *J. Neurooncol.*, **11**, 255–257.

HARRIS EN, GHARAVI AE, ASHERSON RA, BOEY ML AND HUGHES GRV. (1984). Cerebral infarction in systemic lupus erythematosus: association with anticardiolipin antibodies. *Clin. Exp. Rheumatol.*, **2**, 47–51.

HARRIS EN, GHARAVI AE, PATEL SP AND HUGHES GRV. (1987). Evaluation of the anti-cardiolipin antibody test: report of an international workshop held 4 April, 1986. *Clin. Exp. Immunol.*, **68**, 215–222.

HUGHES GRV. (1983). Thrombosis, abortion, cerebral disease and lupus anticoagulant. *Br. Med. J.*, **287**, 1088–1089.

KOZLOWSKI CL, JOHNSON MJ, GORST DW AND WILLEY RF. (1987). Lung cancer immuno thrombocytopenia and the lupus inhibitor. *Postgrad. Med. J.*, **63**, 793–795.

LANG B, NEWSOM-DAVIS J, WRAY D, VINCENT A AND MURRAY N. (1981). Autoimmune aetiology for myasthenic (Eaton–Lambert) syndrome. *Lancet*, **2**, 224–226.

LEGRAND I, LALANDE G, NEUENSCHWANDER S, DULAC O AND KALIFA LG. (1986). Thrombosis of the superior longitudinal sinus in the treatment of lymphoma in children. 4 case reports. *J. Radiol.*, **67**, 595–600.

LEVINE SR AND WELCH KMA. (1987). The spectrum of neurologic disease. Association with antiphospholipid antibodies. *Arch. Neurol.*, **44**, 876–883.

LEVINE SR, DIACZOK IM, DEEGAN MG, KIERAN SN, FEIT H, ELIAS SB AND WELCH KMA. (1987). Recurrent stroke associated with thymoma and anticardiolipin antibodies. *Arch. Neurol.*, **44**, 678–679.

LEVINE MN, GENT M AND HIRSH J. (1988). The thrombogenic effect of anti-cancer drug therapy in women with stage II breast cancer. *N. Engl. J. Med.*, **318**, 404–408.

MAKAR AP, VANDERHEYDEN JS AND VERHEYDEN A. (1990). Maternal and fetal complications associating lupus anticoagulant and its management; three case reports. *Eur. J. Obstet. Gynecol. Reprod. Biol.*, **36**, 185–195.

NASCHITZ JE, YESHURUN D AND LEV ML. (1993). Thromboembolism in cancer: changing trends. *Cancer*, **71**, 1384–190.

MEYRIER A, BECQUEMONT L, WEILL B, CALLARD P AND RAINFRAY M. (1991). Hemolytic–uremic syndrome with anticardiolipin antibodies revealing paraneoplastic systemic scleroderma. *Nephron*, **59**, 493–496.

MILLER DJ. (1967). The association of immune disease and malignant lymphoma. *Ann. Intern. Med.*, **66**, 507–521.

MILLS RC, ZACHARSKI LR AND MCINTYRE OR. (1977). Circulating anticoagulant, autoimmune hemolytic anemia and malignant lymphoma. *Am. J. Med. Sci.*, **274**, 75–81.

MINNA JD AND BUNN Jr. PA. (1989). Paraneoplastic syndromes. In *Cancer: Principles and Practice of Oncology*, 3rd edn. De Vita Jr VT, Hellman S and Rosenberg SA (eds) pp. 1920–1940. J.B. Lippincott: Philadelphia.

MONTALBAN J, CODINA A, ORDI J, VILARDELL M, KHAMASHTA MA AND HUGHES GRV. (1991). Antiphospholipid antibodies in cerebral ischemia. *Stroke*, **22**, 750–753.

PARK CJ, CHO HI AND KIM SI. (1991). A study on changes of coagulation inhibitors and fibrinolysis inhibitors in patients with liver cirrhosis and hepatoma. *J. Korean Med. Sci.*, **6**, 1–6.

RICKLES FR, EDWARDSM RL, BARB C AND CRONLUND M. (1983). Abnormalities in blood coagulation in patients with cancer. *Cancer*, **51**, 301-307.

RIVIER G, TERESA HERRANZ M, KHAMASHTA MA AND HUGHES GRV. (1994). Thrombosis and antiphospholipid syndrome: a preliminary assessment of three antithrombotic treatments. *Lupus*, **3**, 85-90.

SACK Jr GH, LEVIN J AND BELL WR. (1977). Trousseau's syndrome and other manifestations of chronic disseminated coagulopathy in patients with neoplasms: clinical, pathophysiologic and therapeutic features. *Medicine*, **56**, 1-37.

SHAUKAT MN AND HUGHES P. (1990). Recurrent thrombosis and anticardiolipin antibodies associated with adenocarcinoma of the lung. *Postgrad. Med. J.*, **66**, 316-318.

SCHLEIDER MA, NACHMAN RL, JAFFE EA AND COLEMAN M. (1976). A clinical study of the lupus anticoagulant. *Blood*, **48**, 499-509.

SCHREIBER DP AND KAPP DS. (1986). Axillary-subclavian vein thrombosis following combination chemotherapy and radiation therapy in lymphoma. *Int. J. Radiat. Oncol. Biol. Phys.*, **12**, 391-395.

THIAGARAJAN P, SHAPIRO SS AND DE MARCO L. (1980). Monoclonal immunoglobulin M coagulation inhibitor with phospholipid specificity. *J. Clin. Invest.*, **66**, 397-405.

WARREN B AND VALES O. (1972). The adhesion of thromboplastic tumor emboli to vessel wall in vitro. *Br. J. Exp. Pathol.*, **53**, 301-313.

WATTS RA, WILLIAMS W, LE-PAGE S, NORDEN A, SOLTYS A, SWANA G, ADDISON I, HAY FC AND ISENBERG DA. (1989). Analysis of autoantibodies reactivity and common idiotype PR4 expression of myeloma proteins. *Autoimmunity*, **2**, 689-700.

WHISNANT JP. (1990). Special Report from the National Institute of Neurologic disorder and Stroke. Classification of cerebrovascular diseases. *Stroke*, **21**, 637-676.

WISLOFF F, SLETNESS KE AND MICHAELSEN T. (1991). Shared idiotypic determinant in mono- and polyclonal antiphospholipid antibodies with lupus anticoagulant activity. *Thromb. Res.*, **61**, 201-211.